# Novel Insights into the Role of the Mineralocorticoid Receptor in Human Glioblastoma

**DOI:** 10.3390/ijms222111656

**Published:** 2021-10-28

**Authors:** Paula Aldaz, Amaya Fernández-Celis, Natalia López-Andrés, Imanol Arozarena

**Affiliations:** 1 Cancer Signaling Unit, Navarrabiomed, Hospital Universitario de Navarra (HUN), Universidad Pública de Navarra (UPNA), 31008 Pamplona, Spain; paula.aldaz.donamaria@navarra.es; 2 Health Research Institute of Navarre (IdiSNA), 31008 Pamplona, Spain; amaya.fernandez.decelis@navarra.es; 3 Cardiovascular Translational Research Unit, Navarrabiomed, Hospital Universitario de Navarra (HUN), Universidad Pública de Navarra (UPNA), 31008 Pamplona, Spain

**Keywords:** glioma, mineralocorticoid receptor, spironolactone, glucocorticoid receptor, dexamethasone

## Abstract

The majority of glioblastoma (GBM) patients require the administration of dexamethasone (DEXA) to reduce brain inflammation. DEXA activates the glucocorticoid receptor (GR), which can consequently crosstalk with the mineralocorticoid receptor (MR). However, while GR signaling is well studied in GBM, little is known about the MR in brain tumors. We examined the implication of the MR in GBM considering its interplay with DEXA. Together with gene expression studies in patient cohorts, we used human GBM cell lines and patient-derived glioma stem cells (GSCs) to assess the impact of MR activation and inhibition on cell proliferation, response to radiotherapy, and self-renewal capacity. We show that in glioma patients, *MR* expression inversely correlates with tumor grade. Furthermore, low *MR* expression correlates with poorer survival in low grade glioma while in GBM the same applies to classical and mesenchymal subtypes, but not proneural tumors. MR activation by aldosterone suppresses the growth of some GBM cell lines and GSC self-renewal. In GBM cells, the MR antagonist spironolactone (SPI) can promote proliferation, radioprotection and cooperate with DEXA. In summary, we propose that MR signaling is anti-proliferative in GBM cells and blocks the self-renewal of GSCs. Contrary to previous evidence obtained in other cancer types, our results suggest that SPI has no compelling anti-neoplastic potential in GBM.

## 1. Introduction

Glioblastomas (GBM) represent the most frequent form of primary malignant brain tumor, accounting for 50% of cases. GBM affects 1–5 people per 100,000 per year [1,2]. Usually indolent until late stages of progression, the prognosis for malignant gliomas is very poor. Median overall survival from diagnosis is around one year, a situation that has not changed in the last 15–20 years. Post-surgical radiotherapy followed by chemotherapy improves survival by weeks rather than months as tumors invariably regrow [3]. Moreover, the frequent development of tumor and/or therapy associated brain edemas profoundly increases morbidity. Consequently, the administration of anti-inflammatory glucocorticoids, mostly dexamethasone (DEXA) for its low mineralocorticoid activity, is standard of care.

Unfortunately, extensive dexamethasone administration results in unwanted off-target effects [4,5]. Some of them are systemic such as Cushing syndrome, but in recent years there has been an increasing number of reports suggesting that DEXA can affect tumor progression and response to therapies by different mechanisms [5,6]. Recently, we demonstrated that DEXA overrides the mitotic checkpoint pushing GBM cells through the cell cycle [7]. Concurrently, DEXA up-regulates PDGFR signaling, which stimulates expression of anti-apoptotic regulators BCL2L1 and MCL1, required for survival during extended mitosis [7]. Such effects protect GBM cells and stem cells from radiotherapy-induced growth arrest, which emphasizes the controversial role of DEXA in the treatment of GBM.

DEXA exerts its anti-inflammatory effects by binding and activating the glucocorticoid receptor (GR). Typically, active GR translocates to the nucleus to modulate gene transcription (genomic regulation) although GR can regulate by directly binding kinases and other signaling molecules modulating their activity [8]. Physiologically, GR is activated by cortisol. Nevertheless, the duration of GR activation depends on the expression and activity of 11β-hydroxydehydrogenase (HSD11B2), which converts cortisol into its inactive form cortisone.

When expressed in the same cell, GR cross-talks with the mineralocorticoid receptor (MR) at the molecular as well as functional level, and an appropriate balance between GR and MR signaling is crucial for cellular homeostasis. MR binds the mineralocorticoid aldosterone, but can also bind the glucocorticoids cortisol and corticosterone. Glucocorticoids are more abundant than aldosterone, so the activation of MR by aldosterone is dependent on the expression of HSD11B2, which prevents the activation of MR by cortisol. The functions of MR and GR in the same cell type could be complementary or opposing. While the role of aldosterone and MR in the cardiovascular and renal systems has been extensively studied, little is known about MR signaling in cancer, especially on brain tumors [9,10]. Thus, inappropriate MR activation has been shown to promote cardiovascular remodeling in experimental models [11]. Large clinical trials have demonstrated that the addition of MR antagonists (MRAs) to standard care markedly reduced the overall and cardiovascular mortality in patients with heart failure [12].

However, the involvement of MR signaling in the development and/or progression of tumors has not been investigated. MR downregulation predicts poor prognosis in lung cancer and colon cancer [13,14]. Of interest, most of the recent clinical/preclinical findings currently available suggest that MR could be a tumor suppressor [15], although these data need to be confirmed and expanded. Moreover, there are no studies analyzing the effects of MR antagonism in glioblastoma.

With the cross-talk between MR and GR signaling, and the controversial effects of GR signaling when activated by DEXA, we decided to gain insight into MR-dependent signaling in human glioblastoma cells and glioma stem cells (GSCs).

## 2. Results

### 2.1. MR Is Expressed in GBM Cell Lines

**Figure 1:**
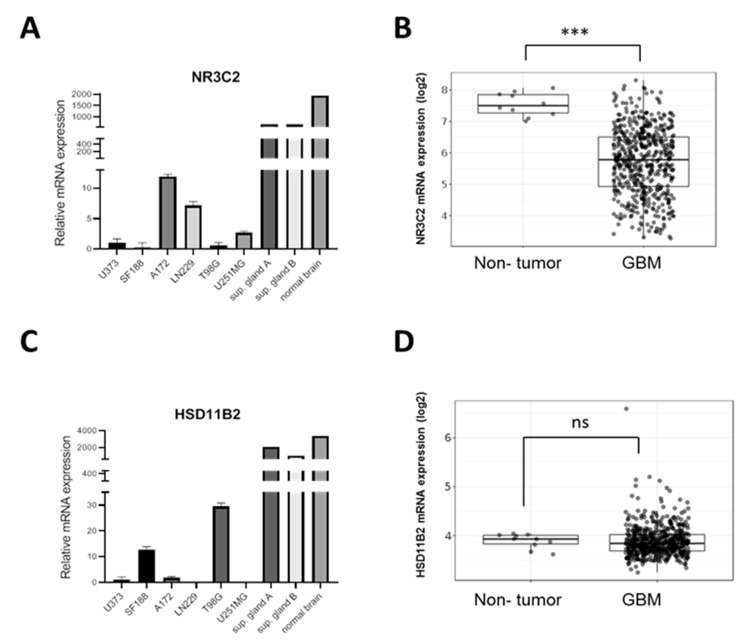
*NR3C2* and *HSD11B2* expression in GBM (**A**) qRT-PCR analysis for *NR3C2* in the indicated cell lines represented as mean fold change of triplicates. (**B**) *NR3C2* (MR) mRNA expression levels in non-tumor (*n* = 10) and GBM (*n* = 528) samples in the TCGA GBM cohort. (**C**) qRT-PCR analysis for *HSD11B2* in the indicated cell lines represented as mean fold change of triplicates (**D**) *HSD11B2* mRNA expression levels in non-tumor and GBM samples in the TCGA cohort. In (**B**,**D**) Tukey’s Honest Significant Difference test was applied (n.s. = not significant, *** *p* < 0.001).

It is well established that cancer cell lines are very heterogeneous, partially due to their genetic instability, often also due to subtype specific transcriptomes. As such, we studied the expression of the *MR* gene *NR3C2* in a panel of glioblastoma cell lines a strictly linear correlation across all lines. As shown in Figure 1A, *NR3C2* expression in a panel of human GBM cell lines was heterogeneous and much lower than in normal human brain or human suprarenal glands. In accordance with our results, analysis of expression data from the TCGA GBM cohort using the GlioVis data visualization tool [16] revealed that *NR3C2* expression is significantly reduced in glioma tumors compared to normal tissue (Figure 1B). Meanwhile, *HSD11B2* mRNA expression levels in GBM cell lines were more heterogeneous. T98G and SF188 cells displayed higher *HSD11B2* levels while LN229 and U251 cells had no detectable levels (Figure 1C). Although there is not a correlation between the levels of the two genes in each GBM cell line, the GBM lines with the highest level of *HSD11B2* (SF188, T98G) are the cell lines with the lowest level of *NR3C2* expression. Unlike *NR3C2, HSD11B2* expression did not significantly change between GBM and normal tumor (Figure 1D).

### 2.2. MR Expression Inversely Correlates with Tumor Progression and Overall Survival

**Figure 2:**
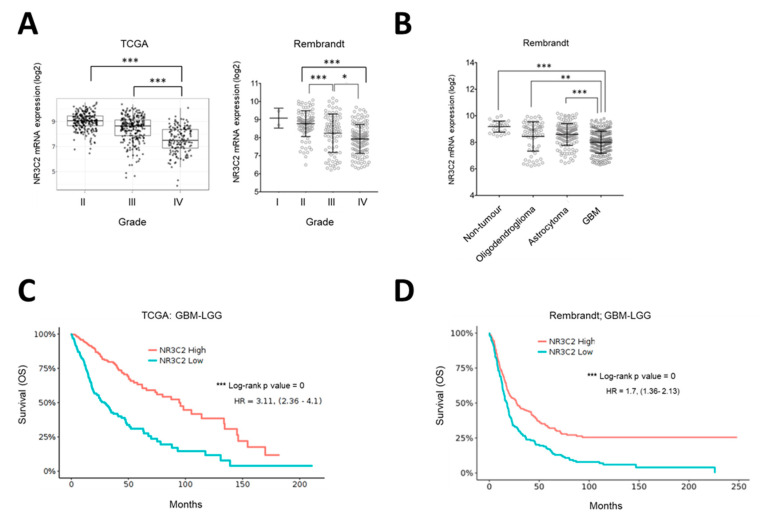
*NR3C2* expression in glioma grades and clinical outcome (**A**) *NR3C2* mRNA expression in TCGA and Rembrandt Glioblastoma datasets in different grades. (**B**) *NR3C2* mRNA expression in low grade glioma subtypes (LGG) and GBM. (**C**) Kaplan-Meier analysis of the GBM-LGG TCGA patient cohort. Differences in overall survival for patients with high (*n* = 333) or low (*n* = 334) expression of *NR3C2* are shown. Hazard ratio and p (log-rank) are indicated. (**D**) Kaplan-Meier analysis of GBM-LGG Rembrandt patient cohort. Differences in overall survival for patients with high (*n* = 201) or low (*n*= 196) expression of NR3C2 are shown. Hazard ratio and p (log-rank) are indicated. * *p* < 0.05, ** *p* < 0.01, *** *p* < 0.001. Datasets were obtained and analyzed using the GlioVis data portal [16].

The fact that *NR3C2* expression was significantly reduced in glioma tumors compared to normal tissue (see Figure 1B) suggested that the expression of the MR was not beneficial for glioma development. Indeed, further analysis of *NR3C2* expression in human glioma tumors showed that its expression significantly diminished with tumor grade (Figure 2A). Indeed, grade IV gliomas (GBM) expressed the lowest levels of *NR3C2*, which could be observed in both the TCGA and Rembrandt cohorts (Figure 2A). Accordingly, GBM samples showed significantly lower *NR3C2* expression than low grade glioma subtypes oligodendroglioma and astrocytoma (Figure 2B). Next, we studied the correlation between *NR3C2* expression and clinical outcomes. The interrogation of patient data including high grade (GBM) and low grade (LGG) gliomas revealed that lower *NR3C2* expression significantly associated with lower overall survival (Figure 2C,D). 

**Figure 3:**
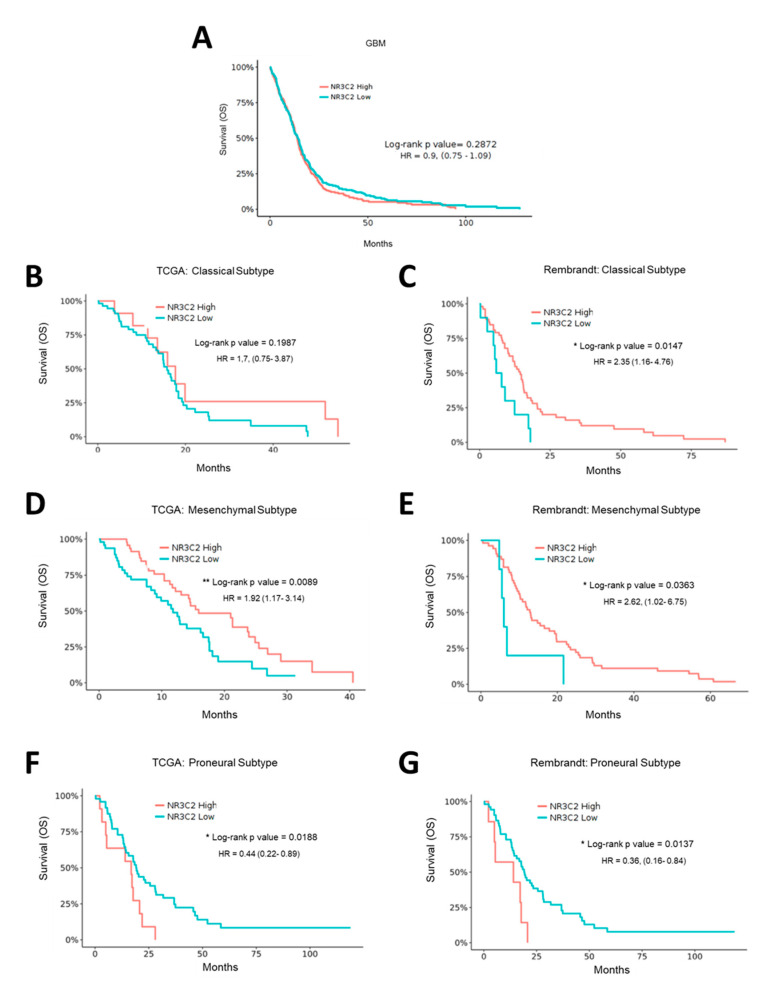
*NR3C2* expression and clinical outcome in GBM subtypes. Kaplan-Meier analysis of (**A**) GBM TCGA patient cohort. Differences in overall survival for patients with high (*n* = 263) or low (*n* = 262) expression of *NR3C2* are shown. (**B**) TCGA Classical subtype patient cohort. Differences in overall survival for patients with high (*n* = 14) or low (*n* = 55) expression of NR3C2 are shown. (**C**) Rembrandt Classical subtype patient cohort. Differences in overall survival for patients with high (*n* = 53) or low (*n* = 10) expression of *NR3C2* are shown (**D**) TCGA Mesenchymal subtype patient cohort. Differences in overall survival for patients with high (*n* = 49) or low (*n* = 49) expression of *NR3C2* are shown (**E**) Rembrandt Mesenchymal subtype patient cohort. Differences in overall survival for patients with high (*n* = 54) or low (*n* = 5) expression of *NR3C2* are shown (**F**) TCGA Proneural subtype patient cohort. Differences in overall survival for patients with high (*n* = 11) or low (*n* = 48) expression of *NR3C2* are shown (**G**) Rembrandt Proneural subtype patient cohort. Differences in overall survival for patients with high (*n* = 7) or low (*n* = 52) expression of *NR3C2* are shown Hazard ratio and *p* (log-rank) are indicated. * *p* < 0.05, ** *p* < 0.01. Datasets were obtained and analyzed using the GlioVis data portal [16].

In grade IV glioma, when omitting subtype specific effects, there was no significant correlation of *NR3C2* expression and patient survival (Figure 3A). However, when we assessed each GBM subtype, we found a correlation between *NR3C2* expression and overall survival. The role of MR in individual subtypes of GBM differs. Intriguingly, while in patients with a GBM of classical or mesenchymal subtype lower *NR3C2* expression correlated with poor survival (in the TCGA ‘classical’ dataset this did not reach statistical significance), the opposite was observed for the proneural subtype (Figure 3B–G). Notably, proneural tumors showed lower *NR3C2* expression than classical or mesenchymal tumors (Supplementary Figure S1). Therefore, that proneural tumors express very low levels of *NR3C2* is in line with the association of longer survival of patients of tumors of the proneural subtype. In summary, our results indicated that *NR3C2* expression is downregulated in more malignant tumors. In LGG, lower expression of NR3C2 is linked to worse survival, suggesting that its down regulation furthers tumor progression. In patients with GBM tumors of the classical or mesenchymal subtype, a reduction of *NR3C2* also correlates with poor survival, whereas in patients with GBM tumors of the proneural type the very low expression of *NR3C2* correlates with better survival. This suggested that the subtype specific molecular make-up of GBM tumors is relevant for the action of MR.

### 2.3. Effect of Aldosterone on GBM Cells and Glioma Stem Cells

The data obtained from the GlioVis analyses suggested that the presence or activation of MR in glioblastoma, only in the classical or mesenchymal subtype, is correlated with better survival. Therefore, we wished to assess the impact of MR activation on cells relevant for GBM.

**Figure 4:**
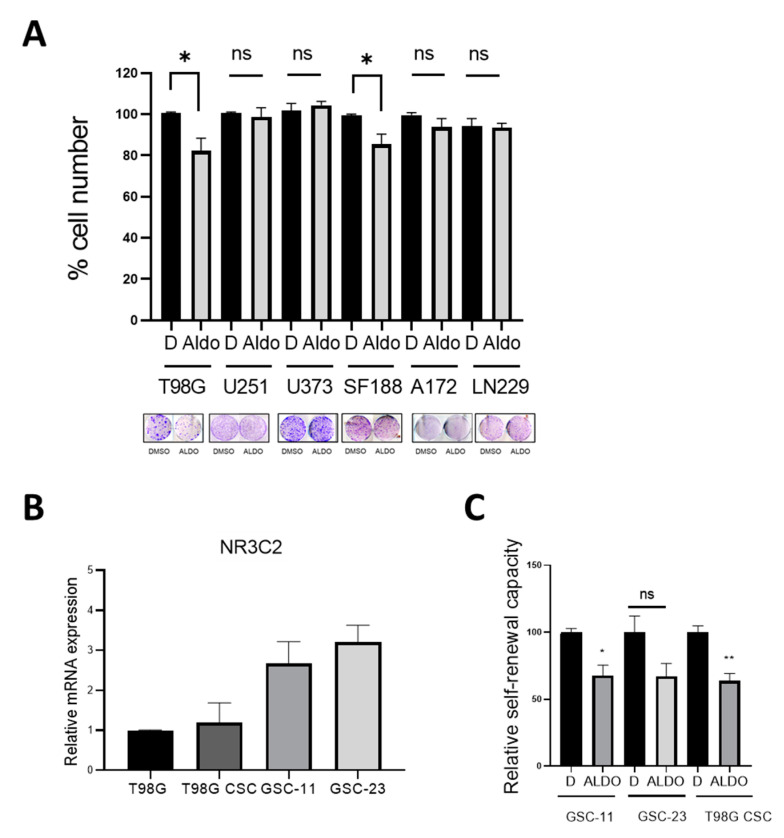
Effect of Aldosterone on glioma cell lines and glioma stem cells (**A**) Quantification of cell numbers in the indicated cell lines in the absence or presence of aldosterone (Aldo) 10^−6^ M. Data represent the mean ± SEM (*n* ≥ 3). A colony formation assay is shown as example. (**B**) qRT-PCR analysis for *NR3C2* in T98G cells, T98G cells grown as neurospheres and GSC-11 and GSC-23 patient derived glioma stem cell lines. Data represented as mean fold change of triplicates. (**C**) Quantification of sphere formation by CSCs cultured in stem cell medium under non-adherent conditions in the absence or presence of ALDO 10^−6^M Data represent the mean ± SEM (*n* ≥ 3). * *p* < 0.05, ** *p* < 0.01, n.s. = not significant.

We first analyzed the effect of aldosterone on growth in six human GBM cell lines. As shown in Figure 4A, physiological concentrations of aldosterone significantly suppressed colony formation of high *HSD11B2* expressing T98G and SF188 cells but no effect on the low *HSD11B2* cell lines A172, LN229, U251, and U373. This suggested that when GBM cells are responsive to aldosterone due to the higher expression of *HSD11B2*, aldosterone has a growth-inhibitory effect. Intriguingly the aldosterone responsive cell lines expressed very low levels of *NR3C2*, whereas *NR3C2* expression levels were variable from high to medium and low in the non-responsive cell lines (see Figure 1A).

GSCs represent a subpopulation of slow-cycling cells with self-renewal capacity characterized by increased radio- and chemotherapy resistance [17]. They have the potential to withstand therapy and promote recurrent tumor growth [18,19,20]. *MR* expression was detectable in T98G-derived CSCs (cancer stem cells) and was comparable to parental T98G cells. In two patient derived GSC cultures (GSC-11 and GSC-23), *MR* expression was slightly increased when compared to T98G cells (Figure 4B). When GSC-11, GSC-23, and CSC-T98G disaggregated cells were treated with aldosterone and left to form neuro-spheroids for 10 days, aldosterone blocked self-renewal in patient derived GSC-cell cultures and in T98G-CSCs, although in these cells it did not reach significance (Figure 4C).

These data suggest that activation of MR by aldosterone inhibits the growth of certain subtypes of GBM cells and suppresses the self-renewal of glioma stem cells, which is in line with the observation that reduced expression of *MR* correlates with poor survival in those subtypes.

### 2.4. Effect of the MR Antagonist Spironolactone on GBM Cells

Our previous data suggested that activation of MR suppresses GBM cell growth. To further assess the role of MR in GBM, we therefore analyzed the effect of spironolactone (SPI) on GBM cell lines. SPI is an MR antagonist with very low affinity for GR that has also been studied as an anti-neoplastic drug [21,22].

**Figure 5:**
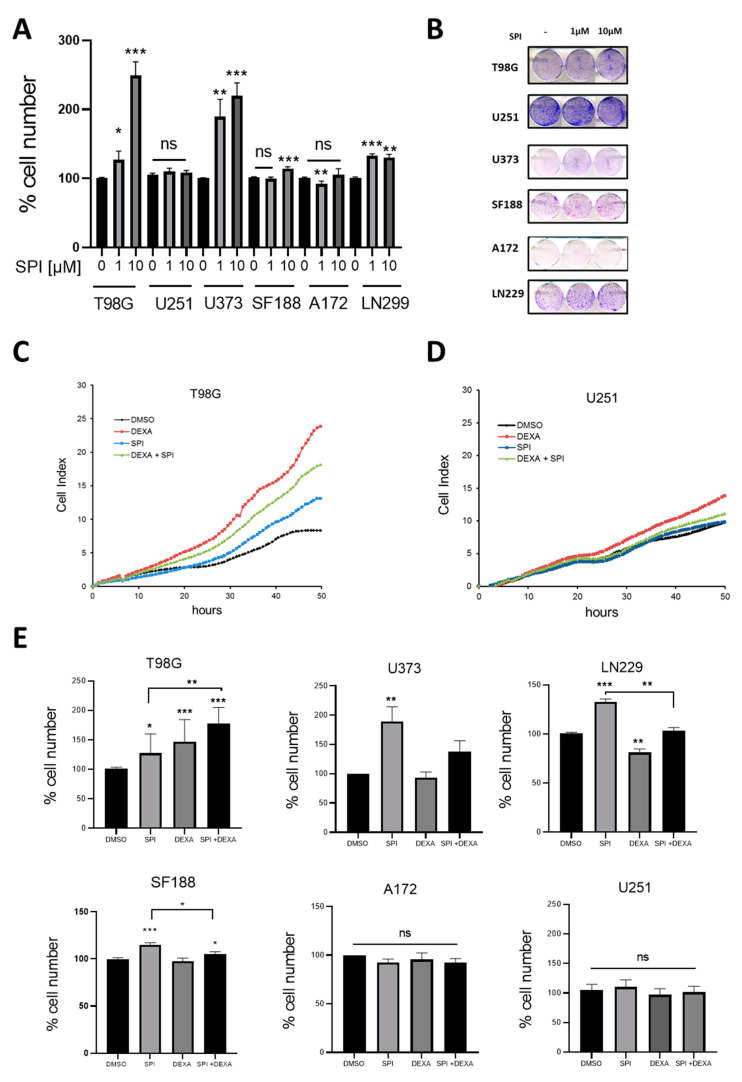
Effect of Spironolactone on glioma cell lines and stem cells (**A**) Quantification of cell numbers in the indicated cell lines in the absence or presence of the indicated concentrations of SPI. Data represent the mean ± SEM (*n* ≥ 3). (**B**) Colony formation assay in glioma cell lines in the absence or presence of the indicated concentrations of SPI. (**C**) xCELLigence™ proliferation analysis of T98G cells treated with DEXA 25 µM, SPI 10 µM and combination of both. (**D**) xCELLigence™ proliferation analysis of U251 as in (**C**). (**E**) Quantification from colony formation assays of cell numbers in the indicated cell lines treated with SPI 1 µM, DEXA 25 µM and combination of both. In (**A**,**E**) data represent the mean ± SEM (n ≥ 3). * *p* < 0.05, ** *p* < 0.01, *** *p* < 0.001.

In line with our previous observations, SPI induced proliferation in T98G, U373, LN229, and SF188 cells. A172 proliferation was slightly reduced by 1uM SPI while U251 cells remained unaffected (Figure 5A,B). In contrast to aldosterone, there was no correlation with the expression levels of *MR* or *HSD11B2* and the effects of SPI in these cell lines.

Next, we assessed the combination of SPI and DEXA treatment on GBM cells. As previously described [7] DEXA alone induced proliferation in T98G and U251 cells, but during the first 48 h of treatment the addition of SPI reduced this pro-proliferative effect of DEXA (Figure 5C,D). However, this inhibitory effect was not seen in long-term survival assays, whereby both DEXA and DEXA+SPI combination provided significant growth signals in T98G cells, but no significant effect was observed in U251 cells (Figure 5E). Furthermore, in U373, LN229, and SF188 cells, where DEXA had no pro-proliferative effect, SPI increased proliferation. No interaction between SPI and DEXA was observed in A172 and U251 cells (Figure 5E).

The effects of SPI appear to be complex. While there is a trend of SPI enhancing GBM cell proliferation, the effect appeared to depend on the cellular background and, as mentioned, was independent of the expression level of *HSD11B2*.

Interestingly, the pro-proliferative dynamics of SPI and DEXA were slightly different. While DEXA-induced proliferation was already detectable 6–12 h after drug addition, the SPI-induced boost of proliferation required around 24 h to be discernible (Figure 5C,D). Given our recent results on the effects of DEXA in GBM cells, we analyzed the mRNA expression of genes regulating the mitotic checkpoint (*TTK, PLK1, TTG1, KIF11*, and *BCL2L1*) or the *PDGFRA* in cells treated with SPI, but no changes were observed (data not shown).

### 2.5. Spironolactone Protects GBM Cells from Radiation

**Figure 6:**
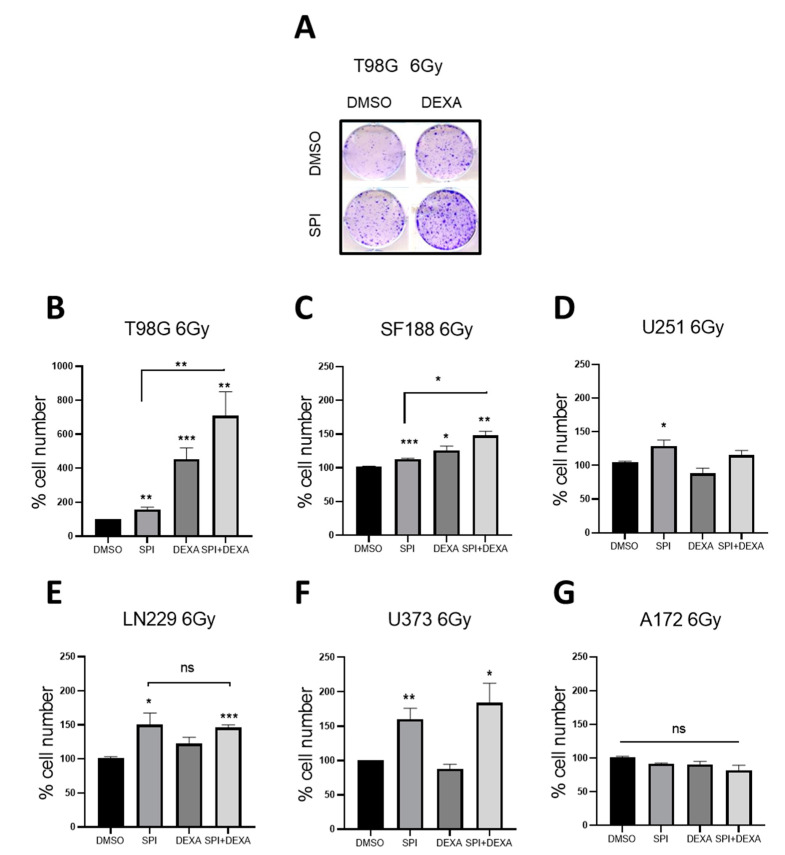
Effects of spironolactone in radiated cells. (**A**) Representative experiment of radiated T98G in the absence or presence of SPI 1 µM, DEXA 25 µM or both. (**B**–**G**) Cell number in the indicated cell lines radiated with 6 Gy and treated with SPI 1 µM, DEXA 25 µM or a combination of both. Data represent mean ± SEM (*n* ≥ 3). * *p* < 0.05, ** *p* < 0.01, *** *p* < 0.001.

As the vast majority of glioma patients undergo adjuvant radiotherapy we next studied the effect of MR inhibition on the response of GBM cells to ionizing radiation. We included DEXA in these experiments because we have shown previously [7] that it can protect cells from radiation. GBM cells were treated with SPI or DEXA 18 h before receiving a single radiation dose of 6 Grays. The cells were analyzed when control cells had reached density. DEXA significantly protected T98G and SF188 cells, but not U251, A172, and U373 cells from radiation. However, in LN229, there was a protective effect that did not reach statistical significance. SPI provided variable but significant protection in all cell lines except A172 cells (Figure 6A–G). Furthermore, the combination of SPI and DEXA induced an additive protection effect to ionizing radiation in T98G and SF188 cells (Figure 6A–C). On the other hand, no additive effects were observed in the other cell lines (Figure 6D–G).

### 2.6. Spironolactone Impairs GSC Self-Renewal

**Figure 7:**
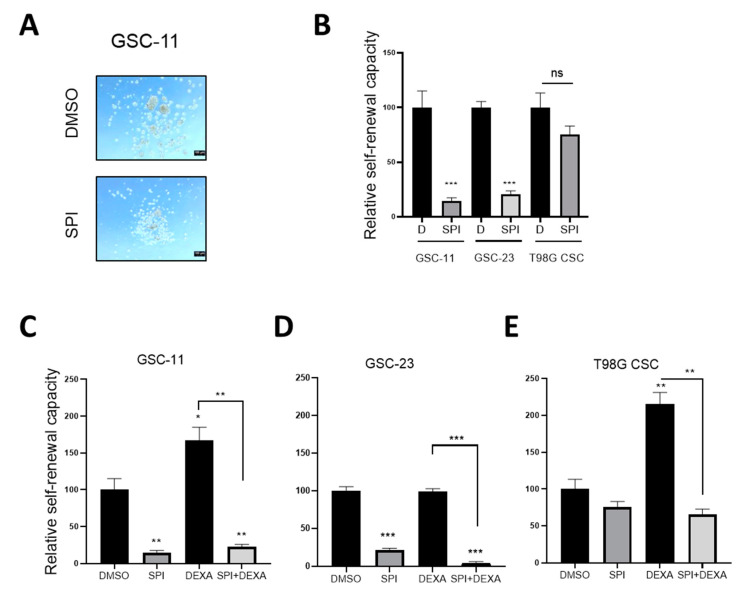
Effects of spironolactone in Glioma Stem Cells (**A**) Images depicting neurospheres formed by GSC-11 under the indicated conditions. (**B**) Quantification of sphere formation by CSCs and GSCs cultured in stem cell medium under non-adherent conditions in the absence or presence of SPI 10 µM. (**C**,**D**) Quantification of sphere formation by the indicated GSCs and CSCs (**E**) cultured in stem cell medium under non-adherent conditions treated with SPI 10 µM, DEXA 10 µM and the combination of both. Data represent mean ± SEM (*n* ≥ 3). * *p* < 0.05, ** *p* < 0.01, *** *p* < 0.001.

We detected an inhibitory effect of aldosterone on the self-renewal of GSCs (see Figure 4C). However, SPI did not induce but rather blocked the self-renewal capacity of GSC-11 and GSC-23. (Figure 7A,B). In CSC-T98G, SPI reduced neuro-sphere formation, but this did not reach significance (Figure 7B).

As we have observed previously [7], DEXA supported stem cell self-renewal in GSC-11, but interestingly not in GSC-23 cultures (Figure 7C,D). In CSC-T98G cells, DEXA stimulated neuro-sphere formation (Figure 7E). In the presence of SPI, self-renewal and neuro-sphere formation were blocked and overcame the stimulating effects of DEXA (Figure 7C,E).

## 3. Discussion

While the effects of glucocorticoids and the GR in cancer have been studied for decades, the role of MR in cancer, especially in gliomas, has barely been addressed. The balance between cortisone and cortisol regulates the activation of both receptors, MR and GR. With the relevance of the GR in DEXA treated GBM, we wished to study the effects of MR activity in glioma cell proliferation. Our results provide new insight into the role of MR in glioblastoma as well as the crosstalk between MR and GR in this cancer type. We observed that activation of the MR by aldosterone inhibited the growth of T98G and SF188 GBM cells. These cell lines show the highest *HSD11B2* expression levels (between 5 and 30 times more than U373 or A172) suggesting that the GBM cell proliferative response to MR activation by aldosterone is dependent on *HSD11B2* expression, when cortisol is present. The medium of GSC/CSC cultures does not contain FCS, but the B27 supplement, EGF and bFGF. It appears that this renders this experimental system independent of *HSD11B2* expression levels.

On the other hand, the MR antagonist SPI induced proliferation in the majority of GBM cell lines analyzed, but this pro-proliferative effect appeared to be independent of *HSD11B2* expression. Activation of the GR by DEXA promotes proliferation by regulating the expression of Spindle Association Checkpoint or mitosis pro-survival genes, such as *MCL1* or *BCL2L1* [7]. However, SPI treatment failed to modulate the expression of these genes (data not shown). Thus, our data suggest a different mechanism of action for DEXA and SPI in promoting cell proliferation. The effects of SPI in cancer cells have been mainly linked to DNA repair mechanisms, whereby it represses homology directed repair and thus sensitizes cells to radiomimetic drugs, PARP inhibitors, or DNA-cross-linking agents [23]. Nevertheless, apart from A172 cells, we observed that, similar to DEXA, SPI induced radioprotection. Moreover, in some cell lines, such an effect was further potentiated when both agents were combined.

Interestingly, SPI’s pro-proliferative effects seen in established GBM cells were no longer observed when their capacity to form stem cell enriched neurospheres was assessed. SPI blocked the self-renewal of GSCs, an effect previously observed in carcinoma stem cells [24]. Furthermore, while DEXA led to increased GSC sphere formation, SPI significantly blocked this activity. Considering the culture conditions for GSCs, with no abundance of aldosterone, the suppressive effect of SPI might rather be due to a mechanism involving the impairment of DNA double-strand break repair as it has been described previously [21]. SPI has been recently identified as a potential drug candidate for a wide range of uses, including tumor immunotherapy or DNA damage-based cancer chemotherapies, suggesting that SPI may be useful in the treatment and prevention of cancers [24]. In light of our data, we believe that such an assumption is not valid for GBM as the effects on established GBM cell lines are opposed to those observed in GSCs.

*MR* expression is lower in glioma than in normal brain tissue and we observe that high *MR* expression strongly correlates with better survival in LGG and classical and mesenchymal GBM. On the contrary, in proneural GBMs, high *NR3C2* expression is linked to worse survival, which could explain the lack of correlation between both variables when analyzing GBMs independently of subtype.

A decrease in MR expression in tumor tissue has been also observed in other tumor types, such as prostate, hepatocellular (HCC), renal, colorectal, and breast cancer [13,25,26,27,28]. Thus, from the expression data in gliomas and previous reports on other cancer types, we could draw the attractive hypothesis of MR acting as a tumor suppressor, although further studies would be required to confirm it. As such, our results with T98G cells would support such a hypothesis although it seems evident that the whole picture must be far more complex, as hinted by our results with GBM cell lines and GSCs. Previous reports have shown that, in HCC, MR expression varied among subtypes, while in colorectal cancer MR expression was reduced independently of stage or differentiation [29]. Amongst GBM patients, proneural tumors correlate with better survival, yet these tumors show reduced *NR3C2* expression rather than classical or mesenchymal ones. Whether the high prevalence of IDH1/2 mutations in proneural tumors or the interplay between MR and specific tyrosine kinase (EGFR, PDGFRs) signaling could explain such an apparent discrepancy warrants further investigation but falls outside of the scope of this study.

## 4. Materials and Methods

### 4.1. Cell Culture and Reagents

T98G (CRL-1690), A172 (CRL-1620), U373MG (HTB-17), and LN229 (CRL-2611) glioma cell lines and HMC3 (CRL-3304) human microglial cells were obtained from ATCC (American Type Culture Collection). U251-MG and SF188 glioma cell lines were a gift from Dr Chris Jones (ICR, London, UK). GBM cell lines were cultured in Dulbecco’s Modified Eagle’s Medium (DMEM) (cat#11995065, Gibco), and HMC3 cells in DMEM/F12 (cat#10565018, Gibco). Human astrocytes (cat#3P10251; Innoprot) were cultured in AM (cat#1801, ScienCell) with supplements (cat#1852, Scien-Cell). GSC-11 and GSC-23 glioma stem cells derived from patients were a kind gift from Dra. Marta Alonso (Solid Tumors and Biomarkers, Foundation for the Applied Medical Research, Pamplona, Spain) and were isolated and cultured as described previously [30]. Human Brain Total RNA (FirstChoice, AM7962) was obtained from Termofisher (Thermo Scientific, Waltham, Massachusetts, USA).

For radiation, cells were irradiated with a Clinac 21EX electron linear accelerator (Varian Medical Systems Inc., Crawley, UK) using a 6MV photon field at a dose of 6 Grays. After a single dose of radiation, cells were left to proliferate as in [7]. Dexamethasone (cat#D4920), Spironolactone (cat#S33785G), and Aldosterone (A94775MG) were obtained from Merck (Darmstadt, Germany).

### 4.2. Patient Data Analysis

Expresssion levels with regard to glioma grade, subtype, and patient survival were analysed using the GlioVis data visualization tool (http://gliovis.bioinfo.cnio.es) accessed on 10 May 2021 [16]. The TCGA and Rembrandt patient data set were interrogated and survival curves created within GlioVis. Expresssion data with regrad tuo tumor grades were extracted and analysed in GraphPad PRISM.

### 4.3. xCELLigence™ Proliferation Analysis

Cell growth and survival were monitored in real time using the xCELLigence Real-Time Cell Analysis system (RTCA DP, ACEA Biosciences-Agilent, Santa Clara, CA, USA) by seeding 5000 cells in the presence or absence of recombinant Dexamethasone.

### 4.4. Colony Formation Assay

Cells seeded in 6-well plates were treated with DEXA (cat#D4902, Sigma) as indicated. If present, spironolactone was added at the same time as DEXA and 18 h before radiation. When control cells had reached density, cells were analysed as described previously [7,31].

### 4.5. Neurosphere Formation Assays

GSC-11, GSC-23, and T98G were grown in NSCs medium for 10 days. Initially formed neurospheres were disaggregated mechanically and enzymatically using accutase (Gibco), and single cells were plated to study secondary neurosphere formation. For quantification studies, 3000 cells were seeded per well of a non-coated 12-well flat bottom plate and fresh media was added every three days. After 10 days, spheres were counted. DEXA and Spironolactone were added 8 h after disaggregation and reseeding and 18 h before radiation.

### 4.6. Real-Time Reverse Transcription PCR

Total RNA was extracted with Trizol Reagent (Qiagen, Hilden, Germany), according to the manufacturer’s instructions. First strand cDNA was synthesized according to the manufacturer’s instructions (Bio-Rad, Hercules, CA, USA). Quantitative PCR analysis was performed with SYBR green PCR technology (Bio-Rad). The following primers were used: MR (F:CCCAACAATTCTGGGCAGAGC, R:ACTCTACCTTCAGCGCAT); 11HSD2 (F:AGAAGCTGCAACAGGTGAC, R:GCGACAGCACTTCTGGATT); h18S (F:GCAATTATTCCCCATGAACG, R:GGGACTTAATCAAGCAAGC); beta-actin (F:GCAAGCAGGAGTATGACGAG, R:CAAATAAAGCCATGCCAATC); GAPDH (F:CAATGACCCCTTCATTGACC; R:GACAAGCTTCCCGTTCTCAG). Quantification of relative gene expression was performed using the AACt method. Data were normalized by h18S and β-actin levels and expressed as a percentage relative to controls. All PCRs were performed at least in triplicate for each experimental condition.

### 4.7. Statistical Analysis

Experimental data are represented as the mean ± SD of three biologic replicates and were compared using Student’s t-test. Significant P-values are indicated with asterisks as follows: * *p* < 0.05, ** *p* < 0.01, *** *p* < 0.001.

## Data Availability

Data are contained within the article or Supplementary Materials.

## Supplementary Materials

The following are available online at https://www.mdpi.com/article/10.3390/ijms222111656/s1, Figure S1: *NR3C2* mRNA expression in different GBM subtypes.
