# Supplementary material for: Novel Insights into the Role of the Mineralocorticoid Receptor in Human Glioblastoma
Source: Int J Mol Sci. 2021 Oct 28;22(21):11656. doi: 10.3390/ijms222111656 (PMC8584062; doi:10.3390/ijms222111656)
Supplement: Supplementary file 1 [file ijms-22-11656-s001.zip › ijms-1419873-supplementary.pdf]

International Journal of
Molecular Sciences

Supplementary Materials

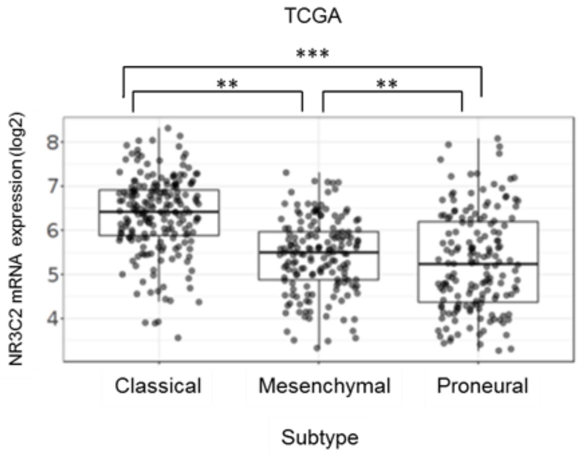

Supplementary Figure S1. ***NR3C2*** **mRNA expression in different GBM subtypes** from TCGA patient cohort. Datasets were obtained and analyzed using the GlioVis data portal [16]. ** $p < 0.01$, *** $p < 0.001$.
